# Classification of BCI Users Based on Cognition

**DOI:** 10.1155/2018/6315187

**Published:** 2018-05-09

**Authors:** N. Firat Ozkan, Emin Kahya

**Affiliations:** Industrial Engineering Department, Eskişehir Osmangazi University, 26480 Eskişehir, Turkey

## Abstract

Brain-Computer Interfaces (BCI) are systems originally developed to assist paralyzed patients allowing for commands to the computer with brain activities. This study aims to examine cognitive state with an objective, easy-to-use, and easy-to-interpret method utilizing Brain-Computer Interface systems. Seventy healthy participants completed six tasks using a Brain-Computer Interface system and participants' pupil dilation, blink rate, and Galvanic Skin Response (GSR) data were collected simultaneously. Participants filled Nasa-TLX forms following each task and task performances of participants were also measured. Cognitive state clusters were created from the data collected using the *K*-means method. Taking these clusters and task performances into account, the general cognitive state of each participant was classified as low risk or high risk. Logistic Regression, Decision Tree, and Neural Networks were also used to classify the same data in order to measure the consistency of this classification with other techniques and the method provided a consistency between 87.1% and 100% with other techniques.

## 1. Introduction

One of the most important issues which ergonomics addresses is the reduction of human errors and accidents. Human errors may have serious consequences from system disruptions to loss of life. Considering that humans have limited capacities both physically and mentally, it is evidently an important necessity to create designs appropriate for human capacity regardless of the task. In-depth studies on human's physical capacity have been performed and are still being performed. For physical fatigue, measurement and calculation methods have been developed, which have general validity in numerous subjects such as muscle fatigue, rest period, and audiovisual capabilities. However, although it is known that humans have limited mental capacities as well, a method determining mental fatigue and mental performance accurately is not available yet. Although there are some accepted methods predicting the cognitive load caused by the task at hand, strengths and weaknesses of these methods are open to dispute.

On the other hand, it is not possible to explain the mental aspect of human errors solely with cognitive load. Although excessive cognitive load is a cause for error, factors which make it more difficult to maintain attention during performance such as extreme stress, mind wandering, and drowsiness due to monotony should also be considered. For this reason, in addition to ongoing studies on measurement of cognitive load, researchers show an interest in the concept of cognitive state as well, which is usually determined with more than one measure.

Within the scope of this study, we performed an experimental study based on the use of some of the methods used in prediction of cognitive load and cognitive state and utilizing BCI systems. BCI systems were utilized for experiments. The idea was to use the ability of giving commands to the computer with brain activities of these systems originally developed for medical purposes and have the participants complete tasks during experiments in this way. 70 students attending Osmangazi University participated in the experiment and completed tasks designed with the Brain-Computer Interface (BCI). Subjective and objective data collected from each participant were evaluated to make a cognitive state classification and a risk identification method was proposed based on instantaneous cognitive states of individuals.

BCI systems have the potential to facilitate life for those who have severely damaged motor skills in accordance with their primary purpose. At this point, all BCI applications, especially Motor Imagery, are beneficial [1]. The use of auditory stimuli based BCI has also been investigated for people who have serious vision disorders besides limited motor skills [2, 3]. The use of BCI systems by healthy people is also a field of interest. It can be said that healthy people are quite successful in using BCI systems especially after training. Healthy participants were found to be highly successful in the BCI experiments for each of the P300, SSVEP, and Motor Imagery brain activities [4–6]. Besides, in an experimental work by Bai et al. [7], participants showed that they could complete the tasks involving typing and selection moves although they spent a long time due to errors. This has led to the researches in using of BCI systems in various nonmedical fields for various purposes. These potential nonmedical areas include device control, user-state monitoring, training/education, safety/security, gaming, cognitive effects of music, and media applications [8, 9]. These findings have been supported by Wang et al. [10] with an experimental study. In their study, the authors designed the Brain-Computer Interface as a cellphone screen and used SSVEP as the potential required for data entry. In the study conducted with ten volunteers, the participants were asked to dial a ten-digit phone number and press the ENTER key on an interface controlled with the SSVEP potential. In another study, Perego et al. [11] applied two different intelligence tests to 19 volunteers. One of these tests was on paper, whereas the other was designed as a Brain-Computer Interface controlled with SSVEP potential. According to the results of the study, there was no significant difference between the BCI-based intelligence test and the on-paper test in terms of performance.

On the other hand, cognitive load detection, cognitive state monitoring, and attention performance are on the forefront of the use of BCI systems in nonmedical areas [8]. It is possible to reach experimental studies that deal with these subjects and some outstanding ones were tried to be summarized.

In their experimental study, Schultheis and Jameson [12] used the P300 potential as an indicator of cognitive load and had 13 participants read texts on a computer screen. After the participants read the text, they were asked 7 questions related to the text. Pupil size and P300 amplitude were measured throughout the experiment. Reading speed of texts with increasing difficulty level was computed as behavioral measures of cognitive load and the participants were asked to evaluate the difficulty level of tasks subjectively. Although reading speed and P300 amplitude indicated a cognitive load as the text difficulty increased, no significant change was observed in pupil size, which was interpreted as an unexpected result in the study.

In their study, Roy et al. [13] stated that BCI systems could be used in cognitive state monitoring and cognitive load determination and conducted an experimental study. The authors designed a BCI task where the participants had to memorize a list of sequential digits visually presented on a screen. Some numbers were missing and the participants had to remember which digit was supposed to be placed in the empty spot in the sequence. The difficulty of the task was set by changing the number of missing digits between 2 and 6. Subjective cognitive load data and task completion times of the participants were considered. It was found that BCI classification performance inclined as the mental fatigue levels of the participants increased.

Chuang et al. [14] designed a BCI system as a driving simulation in their study. It was highlighted in the study that changes in the mental state of the BCI users in the study led to significant activity changes in the occipital region of the brain, that is, the region related to vision, and the mental states of participants were addressed in two classes as alertness/drowsiness. In the study in which various classification methods were used, the Principal Component Analysis (PCA) method showed the highest classification performance with 90%.

Hashemi et al. [15] performed an experimental study with 5 participants and investigated the feasibility of BCI systems to detect instants when the driver feels drowsy. In this study utilizing SSVEP potential, the participants were invited to a laboratory prepared in accordance with conditions inside a car and asked to focus on the target direction according to flickering lights placed on four main directions. It was highlighted in the study that SSVEP potential was highly related to attention level. The Artificial Neural Network method was used to classify mental state, that is, detect instants of drowsiness.

Rozado et al. [16] examined effects of Motor Imagery on pupil diameter. In experiments, the participants were asked to imagine they had performed a grasping motion with their left hands and focus on this thought and then to stop this thought and to not continue the Motor Imagery process, that is, to rest. The participants' pupil dilation was recorded in both states. The results showed that a dilation occurred in pupil during Motor Imagery and a slight constriction occurred during the resting state. These results once again proved that the Motor Imagery is a cognitive process.

Myrden and Chau [17] investigated the relationship between mental state and task performance of participants using BCI. According to the experimental results, BCI performance was 7% lower than average when self-reported fatigue was low and 7% higher than average when self-reported frustration was moderate.

In their study, Lim et al. [18] reported that individuals who had difficulties with using a SSVEP-based BCI technology had lower blink rates and reported higher fatigue.

Although studies on nonmedical use of BCI systems have increased in number in recent years, experimental studies comprehensively using all features of BCI systems have not been conducted yet. From this point, we designed an experimental study containing tasks requiring different brain activities and different cognitive states and requiring individuals to complete tasks with different difficulty levels creating both a drowsiness effect and cognitive loading.

## 2. Materials and Methods

### 2.1. Apparatus

The participants completed tasks using the g.tec BCI system in this study. In addition, a GSR measurement tool and the Tobii X2-60 eye tracker were used in order to measure pupil diameter remotely. For all the tasks, the same 17′′ laptop computer was used.

### 2.2. Participants

Seventy students between the ages of 18–35 participated in the experiment on a voluntary basis. 40 participants were female and 30 participants were male. All of the participants were undergraduate or graduate students at Industrial Engineering Department, Osmangazi University. 64 participants were undergraduate students, whereas 6 participants were graduate students. Also, 9 participants had full- or part-time jobs in addition to their studies. Additionally, 28 participants had an ECTS credit load over 30, 8 participants had a Grade Point Average (GPA) below 2.00, 51 had a GPA between 2.00 and 3.00, and 11 had a GPA of 3.00 or above.

### 2.3. Procedure

It was possible to assess the SSVEP potential as a support to the P300 potential, which is one of the potentials associated with cognitive load most frequently, and the Motor Imagery application with the BCI system used in the study. The following physiological data were measured and recorded simultaneously in order to predict how much difficulty the participants had while completing the tasks by giving commands to the computer with brain activities:

- Change in pupil diameter
- Blink rate
- Galvanic Skin Response

We asked the participants to complete the tasks in BCI applications and conducted classification and prediction studies based on mental loading data during these tasks in order to determine their current cognitive state and performance in tasks requiring attention and mental effort in this state. In experiments, the participants completed three BCI applications twice, each with different parameters and difficulty levels, with 2- to 5-minute rest periods (P300, SSVEP, and Motor Imagery). Participants filled the Nasa-TLX form after each application. Also, signal controls were performed before each BCI application.

### 2.4. Measures

In order to calculate the change in pupil diameter, the average diameter in the first 1 second of the task was used as the base value and the average value in the remainder of the task was proportional to this base value. To find the blink rate, records taken by the eye tracker with 17-millisecond intervals were used. Instants when the data related to eye movements were missing (i.e., the lines were blank) were accepted as instants of blinking and these lines were counted to find the blink rate.

The data normalized with the method proposed by Nourbakhsh et al. [19] were used to calculate the GSR value. To this end, the GSR value in each second of each task was divided into the average GSR value of all tasks completed by the individual in question. Then, these values obtained for each second of the relevant task were summed and divided into task's total length.

## 3. Tasks

**Figure 1:**
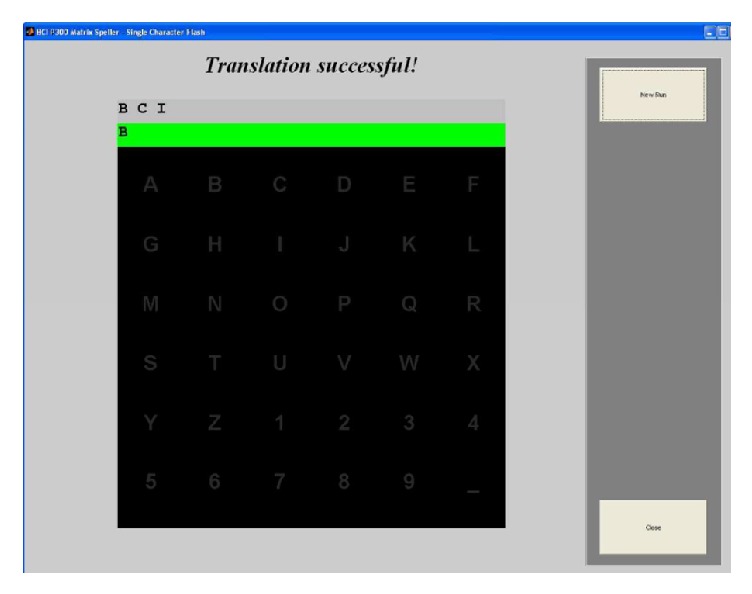
P300 letter matrix.

The BCI system used in the study creates the P300 potential via visual stimuli. A character matrix is used for this and the participants spell words on the screen via this matrix. The spelling of the target character is made possible by the recurring emergence of the P300 potential. Before starting this task, we had the participants spell a random character on the screen initially to familiarize the participants with the matrix. Then, the participants were given the task of spelling two words. The first task to complete in this study was defined as spelling the characters ABCDE, which were side by side on the matrix. During this task, the number of visual stimuli sent by target characters, that is, flashing, was set to 30. The length of visual stimulus was set to 100 milliseconds. Also, the character duplication method was used in this first task. Accordingly, target characters were shown on the screen and the place of the target character was shown to the participants by flashing by itself for a second initially. The free spelling method was used in the second task. In other words, the character to be spelled by the participant was not shown on the screen. Also, flashing count was set to 10 and flashing length was set to 50 milliseconds in order to have the participants make more effort. The array of characters to be spelled on the screen in the second task was determined to be ESOGU2014. This task was explained to the participants only verbally. Thus, the participants had to remember the order of the characters and find the place of the relevant character on the matrix on his/her own.  Figure 1 illustrates a sample screen of P300 application.

**Figure 2:**
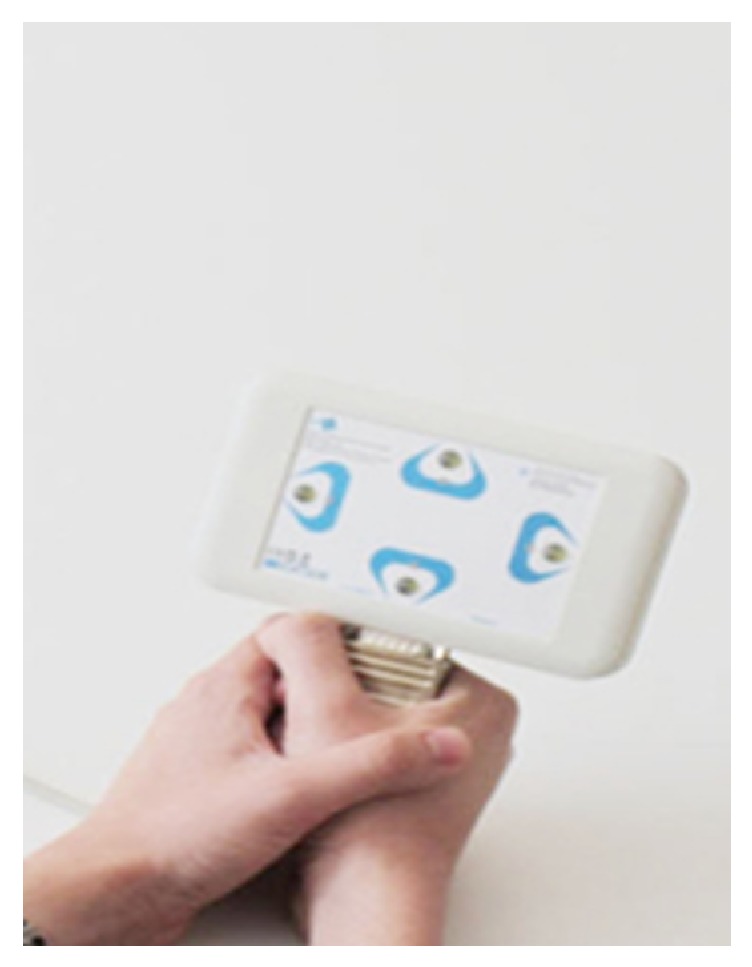
SSVEP control device.

The SSVEP potential occurs by focusing on stimuli with fixed visual frequency. To this end, LEDs representing directions need to flash on a box showing up, down, right, and left and the participants need to follow stimuli coming from the target direction. The participants had to complete two tasks, the first requiring a shorter focus and the second requiring a longer focus. Difficulty levels of the tasks were set by changing the length and interval of the stimuli. The participants focused on LEDs flashing in the order of left, up, right, and down in the first task. There were 10.5 seconds between two directions. In the first task, 70% of these 10.5 seconds were determined as stimulus length and the remaining 30% were waiting period in transitions between two directions. In the second iteration, the visual stimulus length was set to 95% and the transition time was set to 5% to ensure longer focus time and faster transition. SSVEP control device can be seen in Figure 2.

**Figure 3:**
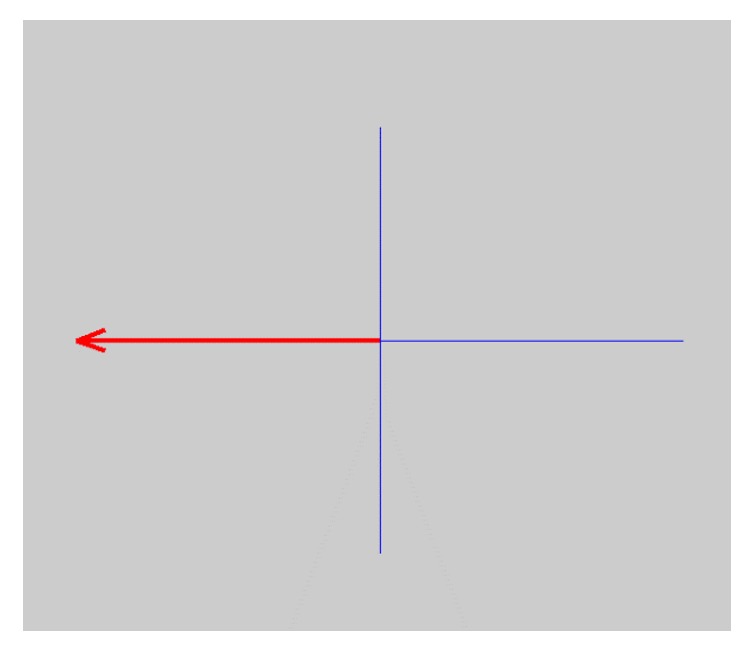
Screenshot of Motor Imagery task.

Essentially, Motor Imagery is an event-related desynchronization/synchronization (ERD/ERS) occurring in the brain. The participants are guided by the *x*-axis on the *x*-*y* plain on the screen. A red arrow appearing on the *x*-axis once in 7 seconds shows the participants which direction (right and left) to focus on. The participants focus on moving the hand on the target direction for 7 seconds; however, they do not make an actual motion with their hands. After 20 iterations in the first task, the second Motor Imagery task started. This time, an additional blue arrow providing feedback to the participants was also present. The blue arrow appearing once the red arrow shows the direction moves to the correct or the opposite direction depending on the participants' focusing success. A screenshot of Motor Imagery task is presented as Figure 3.

The main purpose of the study is to give an opinion about the cognitive state of an individual to complete a task requiring mental effort (thinking, interpreting, calculating, decision-making, etc.) and attention before said task and performance of the individual during said task. A summary of task properties is presented in Table 1.

Nasa-TLX scores were used to evaluate difficulty levels of tasks completed. Nasa-TLX scores showed a normal distribution in at least one normality test and thus were analyzed with paired *t*-test. The results are shown in Table 2 and revealed a significant difference as expected; that is, the second task was more difficult compared to the first task in all task types. SPSS 22 software was used for paired *t*-test analyses.

Task performances of the participants (Table 3) were assessed based on correctly spelled characters in P300 tasks, which involve spelling on a screen. In SSVEP and MI tasks, successful classification percentage during the task was used as the indicator of task performance. The performance classification recommended by g.tec Brain-Computer Interface system consisting of 3 classes (bad-good-excellent) was utilized for interpretations. These performance classes are based on Guger and Edlinger [4], Guger et al. [5], and Guger et al. [6]. In these studies, the success level of individuals in P300, SSVEP, and Motor Imagery tasks was investigated. For each of the 3 task types, at least 50% of the participants achieved 70% classification success and, thus, those who remained below this limit were accepted as low performance, those who achieved a classification success between 70 and 90% were accepted as good, and those who achieved a classification success above 90% were accepted as excellent. Since the aim was to achieve a classification consisting of two classes within the scope of this study, 70% was accepted as the threshold value and below this level was interpreted as low performance.

### 3.1. Proposed Method for Classification of the Participants

Data mining is a collection of methods used to extract important data by analyzing large data sets, which allows for obtaining and interpreting information [20]. It is commonly used thanks to algorithms developed and programs facilitating the use of these algorithms. Classification and clustering are among the most commonly used data mining methods. In classification, classes are predetermined and observations are assigned to appropriate classes within certain rules. Commonly used classification methods include Logistic Regression, Bayes classifiers, Linear Discriminant Analysis, Decision Tree, and Artificial Neural Networks. Clustering is the process of dividing data into groups according to similarity. In general, algorithms which allow for minimizing the distance between the data in the same cluster are used. Clustering analysis may be defined as optimization problems where variables are cluster membership of data points and the objective function is to maximize the similarity between members assigned to these clusters [21]. There are hierarchical and nonhierarchical methods in clustering. The best known hierarchical methods include Nearest Neighbor and Furthest Neighbor algorithms. The *K*-means algorithm can be listed as a nonhierarchical method.

Within the scope of this study, the participants were clustered with the *K*-means method using physiological measurement data. A classification method was proposed taking task performances of participants in these clusters into account. It was expected that the proposed method would be a flexible, multidimensional, and comprehensive tool for BCI studies that can include implementations addressing various cognitive states and brain activities. The steps of the proposed method are as follows:

- Collecting physiological data in order to interpret cognitive state.
- Determining Silhouette scores in order to achieve clusters of 2, 3, and 4 with this data (Table 4).
- Selecting the cluster number with the best score and tagging these clusters by interpreting in terms of cognitive state.
- Dividing task performances into at least two classes.
- Assessing cognitive states and task performances and determining individuals in the positive class in both.

Clusters of 2 were interpreted according to cognitive effort required by the task and cognitive state expected to pose a risk:

- The cluster indicating a higher cognitive loading for P300 tasks, which mostly require cognitive effort, was tagged as the risky cluster.
- Since the main risk was monotony for the SSVEP task due to its nature, the group where physiological indicators opposite to cognitive loading concentrated was tagged as risky.
- Since the cognitive demand was lower and the concentration requirement was higher in the Motor Imagery/1 task, the cluster showing a tendency opposite to cognitive loading was tagged as the risky cluster.
- Since the Motor Imagery/2 task had an indicator providing instantaneous feedback during concentration and followed constantly, which resulted in more sources requiring cognitive effort, the group showing a cognitive loading tendency was tagged as risky.
- In case all physiological indicators supported the interpretation when interpreting clusters, the pupil diameter change was used as base, because it showed significant differences both between different difficulty levels of the same task and between different task types in previous statistical analyses and stood out as the most sensitive physiological measure.

For all tasks, clusters which seemed more risky were tagged with 0 and clusters which seemed less risky were tagged with 1 taking expected physiological outcomes into account (Table 5).

Individuals' cognitive states and task performances were considered to determine task risk classes. The combination of Risk-free Cognitive State-High Performance (70% success at least) was required for the low-risk class. All other combinations were assigned to the high-risk class. In this way, the participants whose cognitive state posed a low risk due to the properties of the task and whose task performance was high in tasks requiring constant attention and concentration were accepted to be low-risk for mental-weighted tasks in general.

Risk classes of all 6 tasks were taken into account in determination of general risk classes. In this classification, the 50% cut-off condition, also used in classification with Logistic Regression, was applied; that is, those who were in the low-risk group for 3 or more of 6 tasks were assigned to the low general risk class. Those who did not meet this condition were assigned to the high general risk class. Thus, 59 out of 70 participants were assigned to the high-risk class, whereas 11 participants were assigned to the low-risk class.

### 3.2. Consistency of the Proposed Method

The consistency of the proposed classification was assessed by comparing the classification to Logistic Regression, Decision Tree, and Artificial Neural Network methods, respectively. The aim of this benchmarking was to confirm the technical validity of the proposed method's steps through different and well-known classification methods that are using various approaches. For Logistic Regression, classification consistency was examined by creating a binary model where physiological indicators and task performance values were used as independent variables in the analysis which was used for classification in this study and employed the ENTER method, which adds all variables to the model at the same time as a block. The cut-off value was 0.50 in the first trial and 0.70 in the second trial. The same classification table formed in both trials and the classification consistency was 100% (Table 7). Model's Nagelkerke *R*^2^ value was found to be 1.0; that is, the model explained the variance in the dependent variable (risk classes) completely. According to the results of the Omnibus test (Table 6), which shows the validity of the model, the model was significant with 95% confidence interval.

Following Logistic Regression, the Decision Tree method was used to test prediction consistency. At this stage, trials were made for CHAID, CART, and QUEST algorithms provided by SPSS 22 package program. In decision trees formed with task performance percentage values and physiological measurement data, the CHAID algorithm provided 90%, the CART algorithm provided 100%, and the QUEST algorithm provided 87.1% prediction accuracy (Table 8).

Following Logistic Regression and Decision Tree, the last method used to test the consistency of the proposed classification method based on cognitive state clusters and task performances was Artificial Neural Networks.

At this stage of the study, a multilayered network structure was used with the Hyperbolic Tangent as the hidden layer activation function and Softmax as the output layer. In this application which used physiological measurements and task performances as input, the entire data was allocated as training set. Trials were made by creating multilayered network structures with Batch, Online, and Mini-Batch training methods included in SPSS 22 package program and the Batch training method provided 100%, the Online training method provided 97.1%, and the Mini-Batch method provided 98.6% prediction accuracy (Table 9).

When the results of the three methods are examined, it can be seen that some differences appeared in the decision points for low-risk class of the methods used. In trials that failed to be 100% consistent, only Artificial Neural Network (Online) method assigned a high risky participant to the low-risk class. When the data of this participant were examined, it was seen that the participant achieved high success in the tasks of P300 and SSVEP compared to average performance. In the case of all the remaining inconsistent classifications, low risky participants were assigned to the high-risk class. When these participants' data were examined, it was observed that their pupil dilations were dominant especially for P300 and SSVEP tasks.

## 4. Conclusion

Errors and accidents caused by human error may occur in every area of life. These errors may have more serious consequences for both human health and performance of the system in areas such as production or transportation. Human error has more than one dimension. These dimensions include the design and conditions of the working environment, physical fatigue, mental fatigue, person's cognitive state, and job's cognitive requirements. The factor which is the most difficult to monitor and detect is person's cognitive state. Cognitive state leads to risks at different levels depending on the job such as excessive cognitive loading, drowsiness due to job's monotony, distraction, and lack of concentration. Most studies in this field focus on detection of drowsiness of vehicle drivers. In addition, various approaches are discussed in studies on detection of cognitive load and mental fatigue.

Although there are methods with an accepted general validity such as Nasa-TLX among subjective methods, the need for stronger tools has encouraged researchers to find new ways. Behavioral measures such as monitoring task performance and monitoring physical indicators related to autonomous nervous system have emerged as alternatives as a result of this search. Particularly physiological indicators related to autonomous nervous system are of great interest due to their objective nature. Physiological methods such as heart rate variability, respiration rate, pupil diameter changes, blink rate and duration, and Galvanic Skin Response stand out in studies on detection of cognitive load and cognitive state. On the other hand, brain activities are preferred to collect data more directly. The fact that developing technology allows not only for tracking brain waves, but also for imaging brain activities in detail has steered studies to this direction. Again with the effect of developing technology, the possibility of tracking a measure with proven sensitivity such as pupil diameter rapidly and accurately provides a great advantage for researchers in this field.

Although the number of methods and devices to use to track physiological indicators and brain activities increases, this does not resolve the complexity of interpreting physiological indicators in particular. Such indicators and activities are affected from environmental factors very quickly, and thus data collection and interpretation are more possible in controlled laboratory environments. In particular, interpreting brain activities requires expertise and it is not easy to use brain activities in studies aimed directly at working life.

At this point, Brain-Computer Interfaces which were originally developed to facilitate the lives of stroke patients and allow for controlling computers and devices with brain activities are of great interest for researchers conducting cognitive studies. These interfaces which can be used with certain special brain activities have begun to be used in cognitive state and cognitive state detection studies in recent years. Detecting whether the relevant brain activity directly occurs via interfaces apart from the signal data means easier interpretation of data provided by these devices.

Six tasks were developed using a BCI system within the scope of this study and data related to pupil diameter, blink rate, and normalized GSR were recorded simultaneously. In this way, we ensured that tasks directly requiring attention and concentration were completed directly with brain activities and advantages of both physiological measurements and behavioral measurements were drawn together. Nasa-TLX, the best known subjective method, was used as supplement.

The data were clustered in later steps. The *K*-means technique was used in clustering analyses and it was found with the help of Silhouette scores that the strongest clustering was obtained when the cluster count was 2. After forming clusters with physiological data of the participants for each task, the clusters were examined individually and the participants' states such as cognitive load, drowsiness, and concentration were interpreted. The participants' expected cognitive state due to the nature of the task and the expected physiological effects of these states were taken into account when making these interpretations.

Once clusters were formed and participants in the negative group and the positive group were tagged for each task, cognitive risk classes were created for each task. At this stage, cognitive states and BCI task performances of the participants were compared. Those who had both a positive cognitive state and a high task performance were assigned to the positive cognitive risk class. Those with negative cognitive state and/or low task performance were assigned to the negative cognitive risk class. Thus, task performances of the participants in tasks requiring attention and their cognitive states when performing these tasks were considered together. General risk classes, on the other hand, were created by seeking a 50% cut-off condition in cognitive risk classes created for 6 tasks. Those who were assigned to the positive cognitive risk class in at least 3 of 6 tasks were assigned to the positive (low-risk) general risk class. Those who did not meet this condition were assigned to high general risk class.

At the next stage, this risk classification was compared to other classification methods. Two trials (cut-off conditions 50% and 70%) with Binary Logistic Regression, the first of these methods, provided 100% consistency. The Decision Tree method, the second of these methods, was tested with 3 different algorithms provided by SPSS 22 package program. One of these algorithms, the CRT algorithm, showed 100% consistency with our classification. The last classification method was the Artificial Neural Networks method. The classification made with the Batch training method by the Artificial Neural Network created with 100% training data provided 100% consistency.

This study constitutes an example for nonmedical use of BCI systems. This experimental method preserving the reliability of brain activities in studies on cognitive state by removing the interpretation difficulty was also supported by physiological indicators. Another advantage of the method was that the tasks performed during the experiment were independent of skill levels of the participants and completed directly with brain activities.

BCI systems are expected to be used much more frequently in studies on cognitive loading and cognitive state in the near future. The flexibility of these systems makes it possible to sector- and purpose-specific interfaces. It is also possible to create authentic simulation environments by integrating BCI systems with three-dimensional virtual environments. Ease of application and interpretation of these systems indicates that they will find their place in working life.

## Figures and Tables

**Table 1:** Task properties and expected physiological effects.

Task	Task properties	Expected main risk factors	Expected physiological effects
P300/1	(i) Creating cognitive load (ii) Constant attention requirement (iii) Intense visual stimulus	(i) Excessive cognitive loading (ii) Increased stress in case of spelling incorrectly	(i) Pupil dilation (ii) Increase in GSR value (iii) Increase in blink rate

P300/2	(i) Creating cognitive load (ii) Constant attention requirement (iii) Very intense visual stimulus	(i) Excessive cognitive loading (ii) Increased stress in case of spelling incorrectly	(i) Pupil dilation (ii) Increase in GSR value (iii) Decrease in blink rate

SSVEP/1	(i) Monotony (ii) Constant attention requirement (iii) Intense visual stimulus	(i) Drowsiness effect	(i) Pupil contraction (ii) Decrease in GSR value (iii) Decrease in blink rate

SSVEP/2	(i) Monotony (ii) Constant attention requirement (iii) Intense visual stimulus	(i) Drowsiness effect	(i) Pupil contraction (ii) Decrease in GSR value (iii) Decrease in blink rate

MI/1	(i) Focusing on a thought (ii) Constant attention requirement (iii) Low intensity visual stimulus	(i) Drowsiness effect (ii) Inability to focus	(i) Pupil contraction (ii) Decrease in GSR value (iii) Decrease in blink rate

MI/2	(i) Focusing on a thought (ii) Constant attention requirement (iii) Constantly following an additional stimulus on the screen	(i) Excessive cognitive loading (ii) Increased stress due to instant feedback	(i) Pupil dilation (ii) Increase in GSR value (iii) Increase in blink rate

**Table 2:** Paired *t*-test results for Nasa-TLX scores.

	Mean	Std. dev.	Std. error mean	%95 confidence interval		*t*	df	*P*
				Lower	Upper			
P 300/1 Nasa-TLX P 300/2 Nasa-TLX	−18.000	13.418	1.604	−21.199	−14.800	−11.223	69	**0.00**
SSVEP/1 Nasa-TLX SSVEP/2 Nasa-TLX	−8.929	9.217	1.102	−11.127	−6.731	−8.105	69	**0.00**
MI/1 Nasa-TLX MI/2 Nasa-TLX	−12.439	10.116	1.209	−14.851	−10.026	−10.287	69	**0.00**

**Table 3:** Task performances (%).

Tasks	Min.	Max.	Avg.	Std. dev.
P 300/1	0	100	56.29	32.36
P 300/2	0	88.89	29.36	25.87
SSVEP/1	53	100	70.99	9.95
SSVEP/2	56	100	71.21	9.25
MI/1	68	85	75.66	3.94
MI/2	70	100	76.76	6.72

**Table 4:** Silhouette scores.

Tasks	Cluster numbers	Silhouette scores	Percentage
P 300/1	**2**	**0.65**	**82.50%**
	3	0.57	78.50%
	4	0.64	82%

P 300/2	**2**	**0.60**	**80%**
	3	0.54	77%
	4	0.59	79.50%

SSVEP/1	**2**	**0.78**	**89%**
	3	0.59	79.50%
	4	0.60	80%

SSVEP/2	**2**	**0.59**	**79.50%**
	3	0.58	79%
	4	0.55	77.50%

MI/1	**2**	**0.59**	**79.50%**
	**3**	**0.59**	**79.50%**
	4	0.58	79%

MI/2	**2**	**0.60**	**80%**
	3	0.52	76%
	**4**	**0.60**	**80%**

**Table 5:** Clustering results.

Tasks	Physiological indicators	Min.	Max.	Avg.	Std. dev.	Cluster center (0)	Cluster center (1)
P 300/1	P 300/1 pupil dilation (%)	−3.72	36.93	11.364	8.75	20.84	5.76
	P 300/1 blink rate per second	0.02	0.3	0.064	0.052	0.08	0.06
	P 300/1 normalized GSR	0.001	0.02	0.005	0.003	47 × 10^−4^	46 × 10^−4^

P 300/2	P 300/2 pupil dilation (%)	−9.96	51.95	23.68	12.604	32.36	11.41
	P 300/2 blink rate per second	0.04	0.407	0.076	0.07	0.07	0.09
	P 300/2 normalized GSR	0.003	0.062	0.006	0.008	58 × 10^−4^	71 × 10^−4^

SSVEP/1	SSVEP/1 pupil dilation (%)	−23.67	50.68	−4.909	12.069	−7.21	32.99
	SSVEP/1 blink rate per second	0.02	0.257	0.056	0.062	0.05	0.15
	SSVEP/1 normalized GSR	0.0003	0.024	0.004	0.003	4 × 10^−3^	85 × 10^−4^

SSVEP/2	SSVEP/2 pupil dilation (%)	−31.75	28.37	−6.395	9.409	−10.55	5.59
	SSVEP/2 blink rate per second	0.017	0.224	0.046	0.04	0.04	0.07
	SSVEP/2 normalized GSR	0.0004	0.022	0.004	0.002	44 × 10^−4^	42 × 10^−4^

MI/1	MI/1 pupil dilation (%)	−23.28	22.16	−1.956	9.026	−7.35	7.76
	MI/1 blink rate per second	0.02	0.48	0.061	0.104	0.04	0.1
	MI/1 normalized GSR	0.001	0.01	0.004	0.002	39 × 10^−4^	41 × 10^−4^

MI/2	MI/2 pupil dilation (%)	−20.62	40.63	2.947	9.589	14.82	−1.48
	MI/2 blink rate per second	0.02	0.35	0.053	0.067	0.07	0.05
	MI/2 normalized GSR	0.001	0.021	0.004	0.002	48 × 10^−4^	4 × 10^−3^

**Table 6:** Omnibus test.

	Chi-square	df	*P*
Step	60.886	24	**0.000**
Block	60.886	24	**0.000**
Model	60.886	24	**0.000**

**Table 7:** Classification results of binary logistic regression.

Observed			Predicted		
			General risk class		Percent correct
			High	Low	
Step 1	General risk class	High	59	0	100.0
		Low	0	11	100.0

**Table 8:** Classification results of decision tree.

	Observed	Predicted		
		High risk	Low risk	Percent correct
*CHAID algorithm*	High risk	59	0	100.0%
	Low risk	7	4	36.4%
	Overall percent	94.3%	5.7%	90.0%

*CART algorithm*	High risk	59	0	100.0%
	Low risk	0	11	100.0%
	Overall percent	84.3%	15.7%	**100.0%**

*QUEST algorithm*	High risk	59	0	100.0%
	Low risk	9	2	18.2%
	Overall percent	97.1%	2.9%	87.1%

**Table 9:** Classification through artificial neural networks.

			Predicted		
			High risk	Low risk	Percent correct
Batch	Training	High risk	59	0	100.0%
		Low risk	0	11	100.0%
		Overall percent	84.3%	15.7%	**100.0%**

Online	Training	High risk	58	1	98.3%
		Low risk	1	10	90.9%
		Overall percent	84.3%	15.7%	97.1%

Mini-Batch	Training	High risk	59	0	100.0%
		Low risk	1	10	90.9%
		Overall percent	85.7%	14.3%	98.6%

## Additional Points

*Practitioner Summary.* Brain-Computer Interfaces are systems in which usage possibilities are investigated in different areas. Experiments using these systems have been carried out to comment on the cognitive status of healthy people in this study. The outputs of the experiments were evaluated through data mining techniques.
